# Self-medication with herbal remedies and pharmaceuticals for COVID-19 among healthcare students in Maputo

**DOI:** 10.1038/s41598-026-53105-2

**Published:** 2026-06-16

**Authors:** Esperança João Rafael, Filomena Barbosa, Saquina Rugunate, Delfina Fernandes Hlashwayo

**Affiliations:** 1 Department of Biological Sciences, Faculty of Sciences, Eduardo Mondlane University, Maputo, Mozambique; 2 Instituto Superior de Ciências e Tecnologia de Moçambique, Maputo, Mozambique

**Keywords:** Self-medication, Healthcare students, COVID-19, Herbal remedies, Conventional medicines, Public health, Infectious diseases, Health care, Medical research

## Abstract

The COVID-19 pandemic triggered widespread self-medication, posing substantial public health risks. Healthcare students represent a critical population to study, as their self-care practices may influence their future professional behavior and patient guidance. This study assessed the prevalence and patterns of self-medication with herbal remedies and conventional medicines for COVID-19 among healthcare students in Maputo, Mozambique. A cross-sectional online survey was conducted in November 2022 among students from Eduardo Mondlane University (UEM), the Higher Institute of Science and Technology of Mozambique (ISCTEM) and the Higher Institute of Health Sciences (ISCISA). Among 390 eligible respondents, 83.6% reported self-medicating for COVID-19: A total of 78.7% used herbal remedies, with 23 plant species and one commercial herbal mixture identified. *Eucalyptus* sp. was the most prevalent (68.2%) used mainly for steam inhalation, followed by lemon (60.5%), ginger (41.3%), garlic (22.1%), and onion (16.2%). Conventional medicines were used by 34.9%, with azithromycin being the most frequent (20.3%). Paracetamol, various antibiotics, and anti-inflammatories were also commonly reported. Notably, 30% of students used both herbal and conventional remedies. The findings suggest the need for targeted educational interventions within healthcare curricula to address potential misinformation, highlight the risks of antimicrobial resistance, and promote evidence-based practices among future health professionals.

**Supplementary Information:**

The online version contains supplementary material available at https://doi.org/10.1038/s41598-026-53105-2.

## Introduction

The COVID-19 pandemic has significantly impacted countries worldwide, with Mozambique facing similar challenges. As a low-income country in sub-Saharan Africa, Mozambique faces high burdens of infectious diseases such as malaria, HIV/AIDS, tuberculosis, and respiratory infections, that place significant pressure to the healthcare infrastructure^1^.

Mozambique experienced considerable health impacts of the COVID-19 pandemic, with a total of 233,891 cases as of February 12, 2025 and 2,252 fatalities^2^. In response to the rapidly evolving crisis, in 2020, the government implemented various measures, including the suspension of in-person classes and restrictions on internal movement. These actions helped delay the peak of the pandemic, reducingpressure on the healthcare system and providing crucial time for the distribution of vaccines and treatments. However, as the virus spread and evidence for effective treatments remained scarce at the outset, many individuals resorted to self-medication, including plants, conventional medicines, and other alternative therapies, to prevent and manage the disease.

Self-medication, particularly during health emergencies when access to evidence-based treatments is limited, is a widespread practice in low- and middle-income countries like Mozambique^3^. This tendency is further intensified by the extensive use of medicinal plants, a deeply rooted aspect of healthcare in many communities^4,5^. Approximately 60% of Mozambique’s population relies on medicinal plants for primary healthcare, particularly in rural areas where healthcare facilities are often remote. Despite the extensive use of these plants^4,6^, there is a lack of standardized knowledge regarding their safety, efficacy, and appropriate dosages. This knowledge gap is concerning, as many individuals erroneously believe that plant-based remedies are inherently safe due to their natural origins. In fact, the improper use of these plants can lead to toxic effects and, in extreme cases, death.

While the World Health Organization (WHO) recognizes the value of traditional, complementary and alternative medicine, it emphasizes the need for scientific validation of these practices^7^. Moreover, the broader issue of self-medication, encompassing the concurrent use of medicinal plants and conventional medicines, remains insufficiently investigated in Mozambique. Studies from other countries have shown that even healthcare professionals and students engage in self-medication^8–10^. With regard to plant-based therapies, individuals often lack the necessary awareness of their pharmacological properties and the risks of interactions with conventional medications. Furthermore, the misuse of antibiotics and other pharmaceuticals during the pandemic has added another layer of concern^8,11^, contributing to antimicrobial resistance and further complicating public health efforts.

In Mozambique, although some plant remedies, such as eucalyptus leaves, were widely used during the COVID-19 pandemic as a form of self-medication^12^, there is limited information regarding the full spectrum of treatments employed, especially among healthcare students. As future professionals, they are expected to be custodians of evidence-based practice and rational pharmacotherapy. However, their medical background and access to pharmaceutical knowledge may paradoxically increase their confidence in self-diagnosis and self-treatment, potentially making them more inclined to self-medicate. Studying their practices offers a unique lens into the perceptions and behaviors that will shape future healthcare systems.

This study examines the use of herbal remedies and conventional medicines for the prevention and treatment of COVID-19 among healthcare students in Maputo. It explores the prevalence and patterns of self-medication and identifies the specific remedies and therapies used. Ultimately, this study aims to inform public health strategies and contribute to a more comprehensive understanding of the role of herbal remedies and/or complementary therapies in public health-seeking behavior during pandemics.

## Results

### Sociodemographic characteristics of study participants

**Fig. 1:**
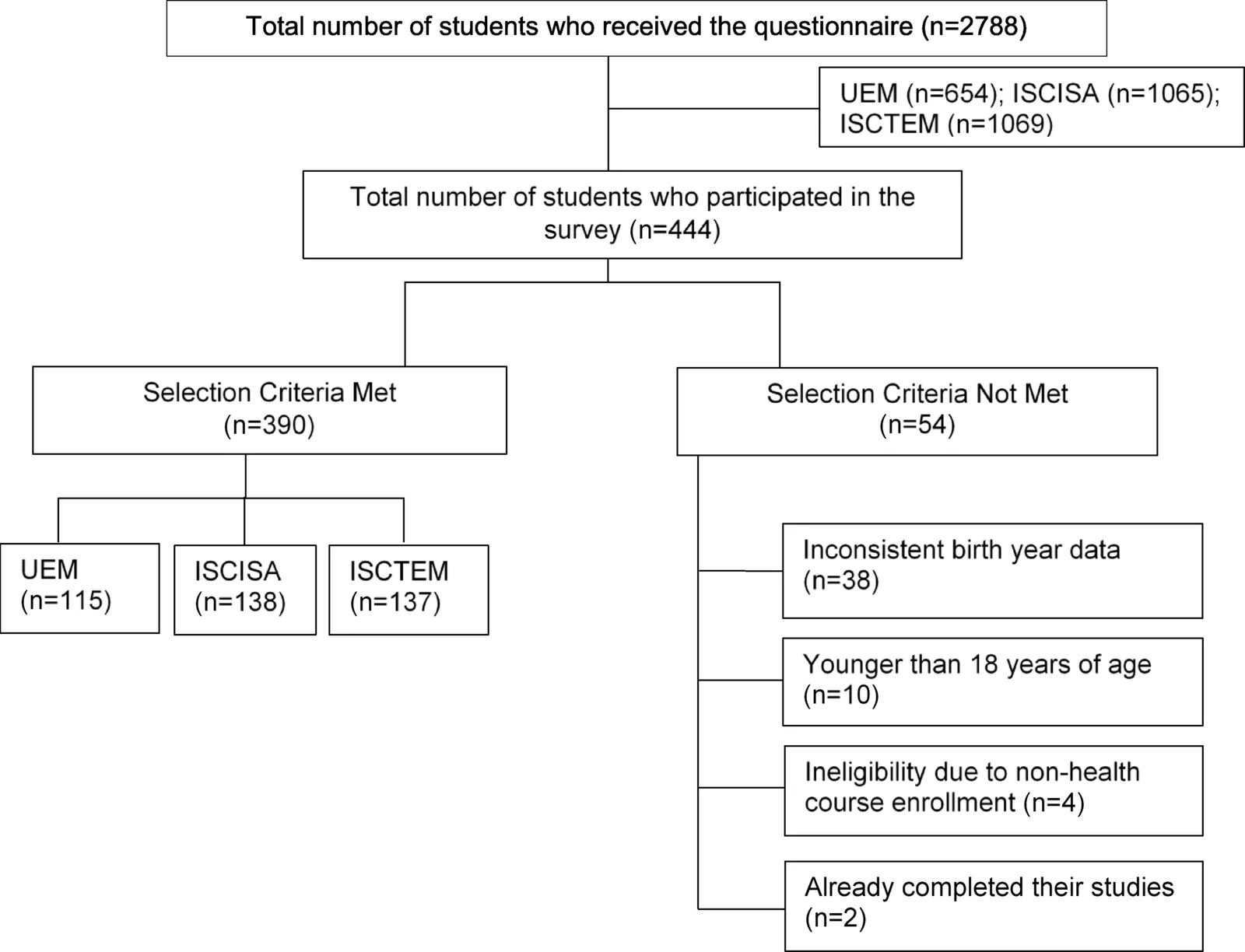
Study participant selection process.

A total of 2,788 students were invited to participate, of whom 444 responded and agreed to participate, yielding a response rate of approximately 16%. A total of 390 of these respondents met the eligibility criteria. Figure 1 illustrates the participant selection process. Among the eligible participants, 29.5% (115) were from Eduardo Mondlane University (UEM), 35.4% (138) from the Higher Institute of Health Sciences (ISCISA), and 35.1% (137) from the Higher Institute of Science and Technology of Mozambique (ISCTEM).

In terms of nationality, 2 participants were foreign nationals (one from Rwanda and one from Portugal). Most participants were female (66.2%, *n* = 258), aged 18–25 years (63.6%, *n* = 248), and single (81.8%, *n* = 319). The most common religion was Christianity (74.4%, *n* = 290). Most participants were undergraduate students (91.3%, *n* = 356). Among respondents to each item, 277 out of 387 were from the southern region of Mozambique, and 78.5% (295/376) were residents of urban areas of Maputo City.

Regarding health status, 11.5% (*n* = 45) of students reported having chronic diseases, and 66.7% (*n* = 260) had experienced symptoms related to COVID-19. A majority (282 out of 389 respondents to this item) had received two or more doses of the COVID-19 vaccine and 107 (27.4%) had a prior diagnose of COVID-19. Detailed sociodemographic information is provided in Table 1.

**Table 1:** Sociodemographic characteristics of study participants.

Characteristic	Category	Frequency (*n*)	Percentage (%)
Gender	Female	258	66.2
	Male	132	33.8
Age (Mean: 25 ± 7.2)	18–25	248	63.6
	> 25	142	36.4
Marital status	Single	319	81.8
	Not single (married/other) †	71	18.2
Religion	Christian	290	74.4
	Muslim	64	16.4
	Others ˫	36	9.2
Nationality	Mozambican	388	99.5
	Other‡	2	0.5
Academic level	Undergraduate	356	91.3
	MSc or PhD students	34	8.7
Year of studies	1st –2nd	206	52.8
	3rd –4th	164	42.1
	5th –6th	20	5.1
Institution	UEM	115	29.5
	ISCTEM	137	35.1
	ISCISA	138	35.4
Geographic region of origin within Mozambique	South	277	71.0
	Centre	60	15.4
	North	50	12.8
	Other§	3	0.8
Had chronic diseases	Yes	45	11.5
	No	291	74.6
	Did not know	54	13.8
Had symptoms of COVID-19	Yes	260	66.7
	No	130	33.3
Prior COVID-19 diagnosis	Yes	107	27.4
	No	283	72.6
COVID-19 Vaccination	1 dose	53	13.6
	2 or more doses	282	72.3
	No vaccination	54	13.8
	Did not answer	1	0.3

†: Not single included: divorced, widowed and married

˫: Others: No religion: n=34, Hindu: n=2

‡: Other nationalities: Portuguese and Rwandese.

§: Two individuals hold foreign nationalities; the province of origin of one participant is unclear.

### Self-medication

Of the respondents, 326 students (83.6%) reported using self-medication for COVID-19 prevention and/or treatment. This included the use of conventional medicines for prevention and herbal remedies for prevention and/or treatment. A total of 117 students (30%) reported using both herbal remedies and conventional medicines.

### Self-medication with herbal remedies for COVID-19 prevention and/or treatment

A total of 307 students (78.7%) reported using herbal remedies for COVID-19 prevention and/or treatment. Of the total sample, 287 (73.6%) used herbal remedies for prevention, 77 (19.7%) for treatment, and 57 (14.6%) for both. The students mentioned a total of 23 plant species, along with one additional commercial herbal mixture composed of various plant extracts. Table 2 provides an overview of used plants, including their scientific and common names, botanical families, plant parts utilized, mode of use, and frequency of citation. *Eucalyptus* sp. was the most frequently cited plant (*n* = 266; 68.2%), with its leaves being primarily used for steam inhalation, although oral consumption in the form of infusions was also reported. Other commonly used plants included lemon (60.5%), ginger (41.3%), garlic (22.1%), and onion (16.2%).

**Table 2:** List of plant species and their uses for COVID-19 prevention and/or treatment as reported by study participants.

No.	Common Name (Scientific Name)	Botanical Family	Parts used	Preparation/Administration	Prevent *n* (%)	Treat *n* (%)	Prevent & Treat *n* (%)	Overall *n* (%)
1.	Eucalyptus (Eng.), Eucalipto (Pt.) (*Eucalyptus* sp.)	Myrtaceae	Leaf	Steam †‡ (inhalation), infusion †(oral)	244 (62.6)	72 (18.5)	50 (12.8)	266 (68.2)
2.	Lemon (Eng.), Limão (Pt.) (*Citrus limon* (L.) Osbeck)	Rutaceae	Leaf, fruit	Steam †‡ (inhalation), infusion †‡, add to cold water †, syrup †, juice ‡ (oral)	216 (55.4)	59 (15.1)	39 (10)	236 (60.5)
3.	Ginger (Eng.), Gengibre (Pt.) (*Zingiber officinale* Roscoe)	Zingiberaceae	Rhizome	Infusion †‡, syrup ‡ (both oral)	139 (35.6)	50 (12.8)	28 (7.2)	161 (41.3)
4.	Garlic (Eng.), Alho (Pt.) (*Allium sativum*)	Amaryllidaceae	Bulb	Raw †, infusion †‡, syrup ‡ (all oral)	69 (17.7)	34 (8.7)	17 (4.4)	86 (22.1)
5.	Onion (Eng.), Cebola (Pt.) (*Allium cepa* L.)	Amaryllidaceae	Bulb	Syrup †‡, infusion ‡ (both oral)	53 (13.6)	19 (4.9)	9 (2.3)	63 (16.2)
6.	Carrot (Eng.), Cenoura (Pt.) (*Daucus carota* L.)	Apiaceae	Root	Syrup †‡ (oral)	25 (6.4)	7 (1.8)	4 (1)	28 (7.2)
7.	Guava (Eng.), Goiaba (Pt.) (*Psidium guajava* L.)	Myrtaceae	Leaves	Infusion †(oral), decoction ‡ (massage on lower abdomen)	10 (2.6)	4 (1)	0	14 (3.6)
8.	Mango tree (Eng.), Mangueira (Pt.) (*Mangifera indica* L.)	Anacardiaceae	Leaves	Steam †(inhalation)	4 (1)	1 (0.3)	0	5 (1.3)
9.	Turmeric (Eng.), Acafrão (Pt.) (*Curcuma longa* L.)	Zingiberaceae	Rhizome	Infusion †, syrup ‡, mixed with hot milk and honey † (oral)	4 (1)	3 (0.8)	3 (0.8)	4 (1)
10.	Boldo (Eng. and Pt.) (possibly *Peumus boldus* Molina or *Coleus barbatus* var *barbatus*)§	Monimiaceae / Lamiaceae	Leaves	Infusion † (oral)	2 (0.5)	0	0	2 (0.5)
11.	Cactus (Eng.), Cacto (Pt.) (*Opuntia ficus-indica* (L.) Mill.)	Cactaceae	Stem	Syrup ‡ (oral)	2 (0.5)	0	0	2 (0.5)
12.	Cinnamon (Eng.), Canela (Pt.) (*Cinnamomum* sp.)	Lauraceae	Stem bark	Herbal paste ‡ (oral)	2 (0.5)	0	0	2 (0.5)
13.	Lemongrass (Eng.), chá príncipe (Pt.), capim-limão (Pt.) (*Cymbopogon citratus* (DC.) Stapf)	Poaceae	Leaves	Steam † (inhalation) / Infusion ‡ (oral)	2 (0.5)	0	0	2 (0.5)
14.	Black pepper (Eng.), Pimenta preta (Pt.) (*Piper nigrum* L.*)*	Piperaceae	Fruit	Herbal paste ‡ (oral)	1 (0.3)	0	0	1 (0.3)
15.	Cloves (Eng.), Cravinho (Pt.) (*Syzygium aromaticum* (L.) Merr. & L.M.Perry)	Myrtaceae	Flower bud	Herbal paste ‡ (oral)	1 (0.3)	0	0	1 (0.3)
16.	Cidreira (Pt.) (*Lippia javanica* (Burm.f.) Spreng.)	Verbenaceae	Leaves	Infusion † (oral)	1 (0.3)	0	0	1 (0.3)
17.	Mafurreira (Loc.) (*Trichilia emetica* Vahl)	Meliaceae	Leaves	Decoction ‡ (massage on lower abdomen)	1 (0.3)	0	0	1 (0.3)
18.	Orange (Eng.), Laranja (Pt.) (*Citrus × sinensis*)	Rutaceae	Fruit	Juice ‡ (oral)	0	1 (0.3)	0	1 (0.3)
19.	Mapfilwa (Loc.) (*Vangueria infausta* Burch.)	Rubiaceae	Leaves	Decoction ‡ (massage on lower abdomen)	1 (0.3)	0	0	1 (0.3)
20.	Flor de Liz (Pt.) (Scientific name not identified)	Unidentified	Flower	Herbal paste ‡ (oral)	1 (0.3)	0	0	1 (0.3)
21.	Yam (Eng.), Inhame (Pt.) (*Dioscorea* spp.)	Dioscoreaceae	Tuberous root	Yam milk † (blended with warm water)	1 (0.3)	1 (0.3)	1 (0.3)	1 (0.3)
22.	Mint (Eng.), Hortelã (Pt.) (*Mentha spicata* L.)	Lamiaceae	Leaves	Infusion † (oral)	1 (0.3)	0	0	1 (0.3)
23.	Okra (Eng.), Quiabo (Pt.) (*Abelmoschus esculentus* (L.) Moench)	Malvaceae	Fruit	Unspecified †	1 (0.3)	0	0	1 (0.3)
*	Lianhua Qingwen Keli (Formulation)*	Commercial herbal mixture		Infusion (oral)	1 (0.3)	0	0	1 (0.3)

§ The term “boldo” may refer to either *Peumus boldus* (Monimiaceae) or *Coleus barbatus* var. *barbatus* (Lamiaceae), both of which are commercially available. However, it was not possible to determine which species participants used in this study.

† Used alone, ‡ Used in combination with other plants, †‡ Used both alone and in combination.

* This is a commercial traditional Chinese herbal formulation composed of multiple plant extracts.

Eng. (English), Pt. (Portuguese), Loc. (Local language)

Participants reported various plant combinations used for COVID-19 prevention and/or treatment. In some cases, the same plant was used in multiple preparations, such as infusion, syrup, steam inhalation and other therapeutic preparations.

Among students who reported using herbal remedies for COVID-19 prevention, 179 indicated a duration of use of seven days or more, including 26 who reported use for up to one year. A total of 138 students reported daily use. Among those who used herbal remedies for the treatment of COVID-19, 54 students rated their impact on recovery as high, giving scores of four or five on a scale from zero to five.

Most students reported obtaining information about herbal remedies from multiple non-medical sources, most frequently from family, friends or close relatives (*n* = 238), followed by social media (*n* = 93). Other sources, such as television, internet searches, health professionals, newspapers, scientific articles, books, and others, were less commonly mentioned. Notably, a small number of students (*n* = 6) reported obtaining information from traditional healers.

Of the students who used herbal remedies to treat COVID-19, only five reported experiencing side effects, including abdominal pain, vomiting, sore throat, and dizziness. Although a clear causal relationship could not be established, the herbal preparations ingested or inhaled by these individuals included turmeric tea, guava leaf tea, ginger tea (alone or combined with garlic), lemon tea, steam inhalation of eucalyptus leaves (alone or mixed with lemon leaves), steam inhalation of lemon leaves alone, and onion syrup. Due to the frequent simultaneous use of multiple remedies, it was not possible to attribute each effect to a specific preparation.

### Self-medication with medicines to prevent COVID-19

A total of 136 students (34.9%) reported using conventional medicines to prevent COVID-19. Fourteen different medicines were mentioned, with Azithromycin being the most frequently used, reported by 79 students (20.3% of the total sample, 58.1% of those using conventional medicines). Among these students, 54 reported using more than one type of medicine: 28 mentioned two medicines, 20 mentioned three, and six mentioned four. The most frequent combination was Azithromycin and Paracetamol, reported by 13 students.

**Fig. 2:**
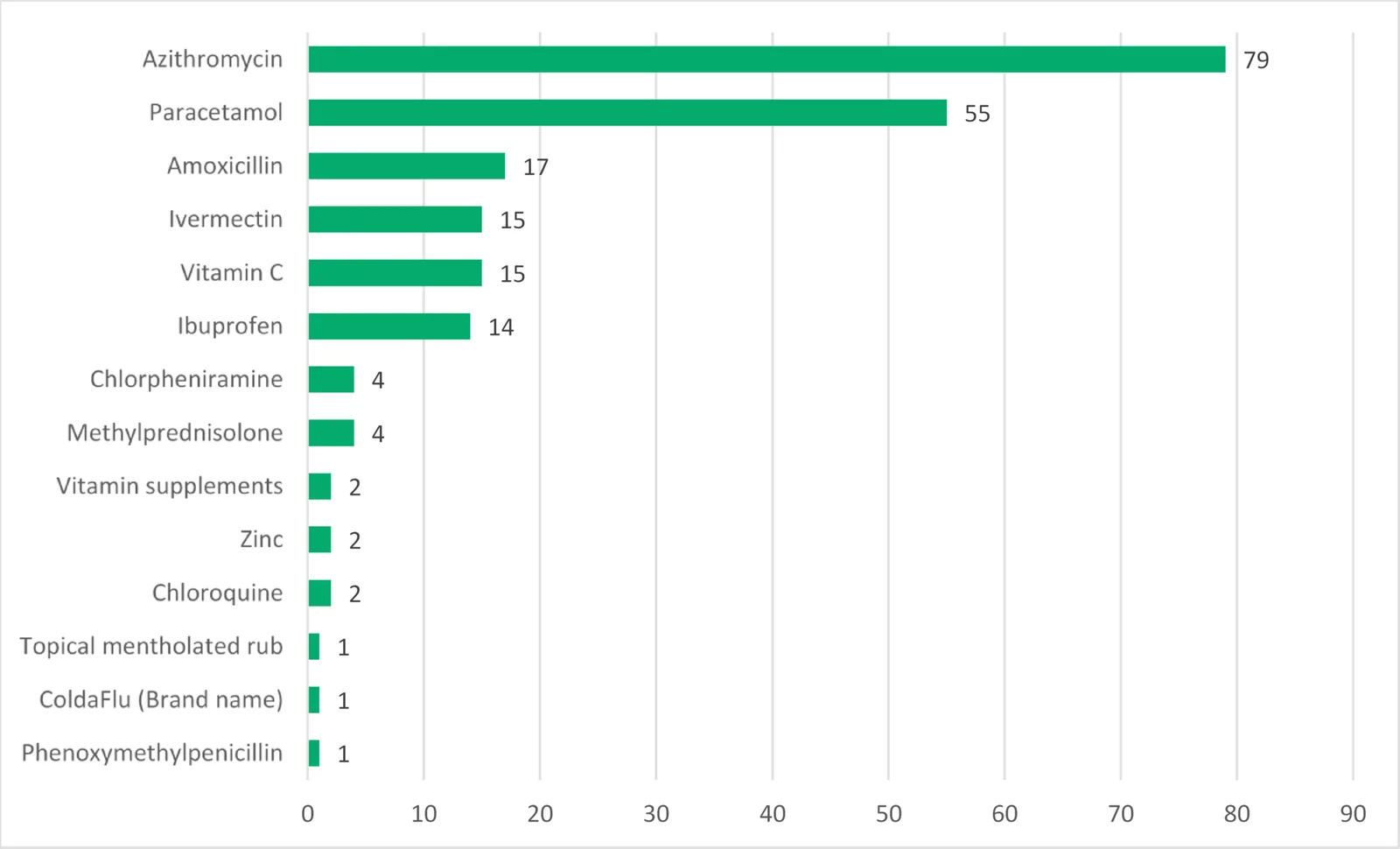
Medicines used for COVID-19 prevention and their respective percentages.

**Fig. 3:**
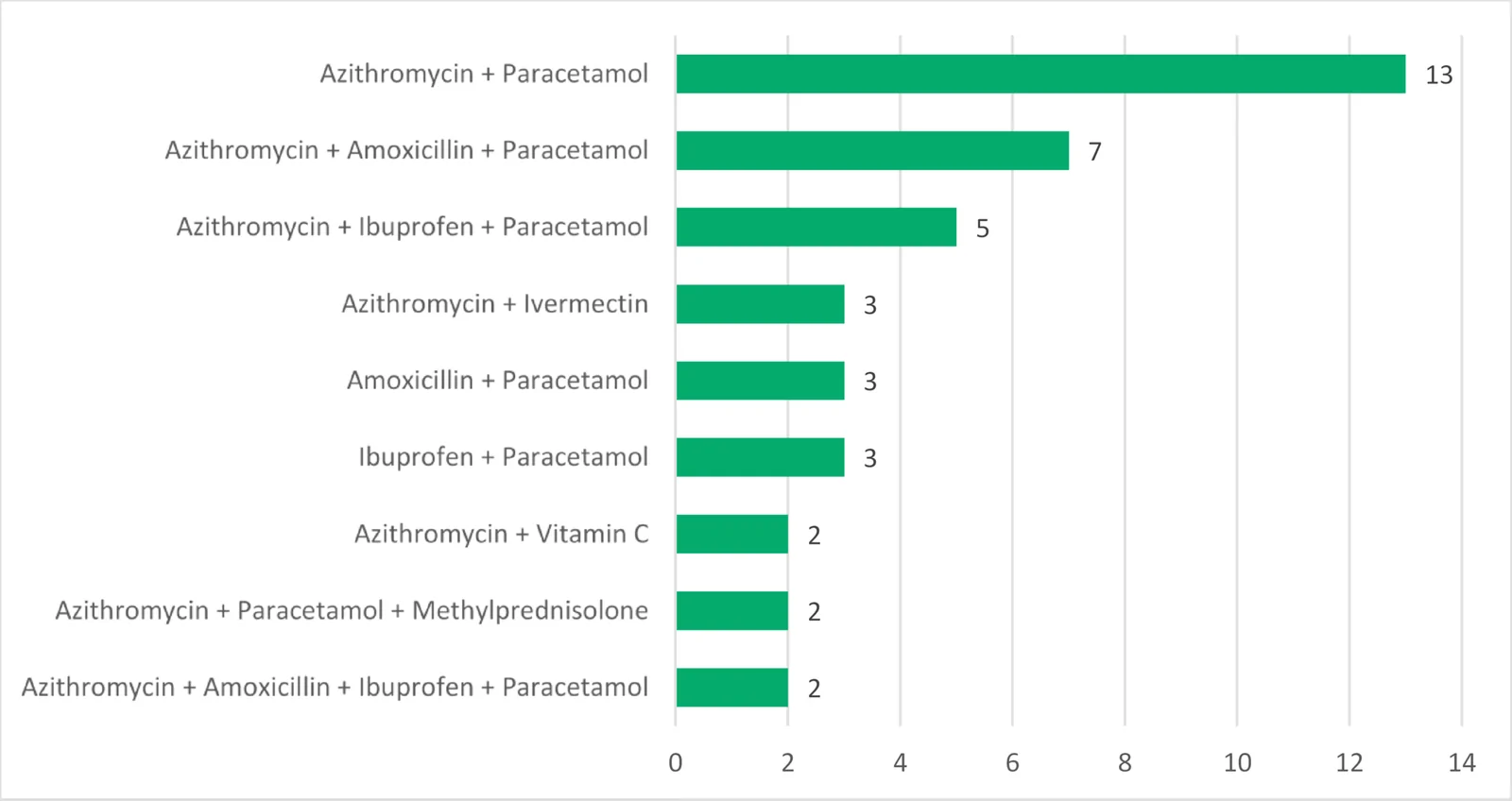
Multiple medicines reported by students for COVID-19 prevention and number of students reporting each [*Only combinations reported by two or more students are shown*].

Among all therapeutic classes mentioned, antimicrobials were the most frequently mentioned (*n* = 114), predominantly antibiotics (*n* = 97). Anti-inflammatory and analgesic agents followed (*n* = 73), along with nutritional and mineral supplements (*n* = 19), antihistamines (*n* = 4) and nasal decongestants (*n* = 2). Figure 2 presents the absolute number of mentions for each medication used, while Fig. 3 shows the frequency of multiple drugs reported by two or more students. Other multiple-drug reports, each mentioned by a single student, are listed in the study database.

## Discussion

This study highlights a high prevalence of self-medication among study participants (83.6%), with a particular emphasis on the use of herbal remedies to prevent and/or treat COVID-19, especially *Eucalyptus*, which was the most used plant. Despite being enrolled in health-related programs, primarily in the medical field, most of these students receive no formal training in phytotherapy, and these results may reflect reliance on traditional medicine and sociocultural factors on their health-seeking behaviors. With regard to conventional medicine use, the widespread and unregulated use of antibiotics raises serious public health concerns, particularly regarding adverse effects^13^ and antimicrobial resistance^14^.

This study also reveals a varied pattern of self-medication among healthcare students. While some relied solely on herbal remedies and others on conventional medicines, a subset reported using both, reflecting the coexistence of traditional and biomedical approaches even among individuals receiving formal health training. This heterogeneity is further evident in the range of agents employed, from single to multiple plant species and conventional medicines, and in the highly variable durations of use. While the specific sequencing (concurrent versus sequential use) remains unclear, these patterns collectively suggest a pragmatic, condition-driven approach to self-care that operates outside standardized therapeutic protocols. Notably, the sources of information guiding these practices extend beyond formal education to include familial networks, social media, traditional healers and other reported sources. Although a minority, the consultation of traditional healers shows that even future health professionals recognize the importance of Indigenous knowledge systems.

The prevalence of herbal remedy use in this cohort aligns with observations from studies conducted in other countries such as Pakistan^11^, Peru^15^, and Jordan^16,17^ and Morroco^18^. While no studies in Mozambique have specifically explored the use of herbal remedies among health students for COVID-19, a study in the Montepuez district (Cabo Delgado province), Mozambique reported that 27.8% of residents used herbal remedies to treat COVID-19^19^. The higher proportion observed in our study may reflect the influence of geographic, demographic, and cultural factors on self-medication practices. These findings highlight the need for health-related academic programs in Mozambique to enhance their curricula by including specific training on the risks of self-medication and the safe integration of phytotherapy with conventional treatments. This approach would promote evidence-based and culturally responsive healthcare while preparing future professionals to navigate both traditional and modern therapeutic options safely. Furthermore, it is important to conduct further research on the use of complementary and alternative therapies in Mozambique to better understand the extent of their use and implications for public health.

*Eucalyptus* sp. was the most used species for the prevention and treatment of COVID-19 among the students. This finding is consistent with those of a study conducted in Peru, where most respondents reported using *Eucalyptus* to manage COVID-19^15^. Furthermore, this finding aligns with the well-established role of *Eucalyptus* in traditional medicine in various countries, particularly for respiratory conditions^15,20^. During the COVID-19 pandemic, demand for *Eucalyptus* increased significantly in Maputo city, Mozambique, leading to noticeable defoliation of trees due to its widespread informal use for steam inhalation. While *in vitro* and *in silico* studies have highlighted antiviral properties and anti-inflammatory effects of eucalyptus^21–23^, the absence of standardized dosages raises safety concerns.

Other commonly used plants included lemon (*Citrus limon*), ginger (*Zingiber officinale*), garlic (*Allium sativum*) and onion (*Allium cepa*), which are known for their immune-supporting properties^24–26^ and were frequently prepared as infusions, steam inhalations and syrups. The use of lemon and ginger as home remedies for COVID-19 has been widely reported in other studies^18,27^. Although many of the plants used by students have been investigated for their potential effects against COVID-19^23,28–30^, current evidence remains variable and, in many cases, inconclusive. The use of these products should be guided by clear and evidence-based guidelines. This is particularly important for individuals with pre-existing health conditions or weakened immune systems, who may be at greater risk of complications from unregulated self-medication. Establishing and disseminating clear guidelines is essential to ensure that traditional knowledge is applied safely and consistently with scientific evidence.

In this study, participants reported perceived benefits associated with the use of plants. However, such perceptions should be interpreted with caution, as they represent subjective experiences rather than objectively verified clinical outcomes.

The mild side effects reported by a small portion of students in this study are consistent with findings from studies in Jordan , where health students and professionals also reported minor adverse effects from herbal remedies including nausea, vomiting, and diarrhea^17^. Although generally mild and transient, these symptoms challenge the perception of absolute safety often associated with herbal medicines. They may result from unstandardized dosages, variable preparation, or individual sensitivities, and can pose increased risks for immunocompromised individuals. These findings highlight the need for pharmacological research to define safe therapeutic windows.

Self-medication with conventional medicines was commonly reported among study participants. Although it is a documented phenomenon in Mozambique^3,31,32^, to the best of our knowledge, this is the first study to specifically assess its prevalence among health students in Maputo. Similar findings have been observed globally. For instance, a study conducted at Aga Khan University in Karachi, Pakistan^33^, found that 76% of medical and non-medical university students engaged in self-medication. Promoting the rational use of pharmaceuticals is essential to prepare informed professionals committed to safe and evidence-based practices. As future healthcare providers, these students should be acutely aware of the dangers of self-medication practices and antibiotic misuse, including the increased likelihood of inadequate therapy, delays in receiving proper treatment, development of antimicrobial resistance, and morbidity^34^. The frequent use of azithromycin and paracetamol observed in this study aligns with reported widespread use of azithromycin for COVID-19 prevention. While paracetamol use, although potentially excessive, aligns with symptom management, the frequent use of azithromycin, is concerning. Its widespread use, fueled by misinformation and initial speculative hypotheses, poses a serious public health threat by accelerating antimicrobial resistance and potentially compromising treatment of common bacterial infections in the future. Unlike herbal remedies, which primarily carry individual risks, the misuse of antibiotics creates a collective, public health and ecological threat. These findings underscore the urgent need for targeted public health interventions to correct misconceptions about antibiotics, reinforce regulatory measures against over-the-counter sales, and communicate the broader community risks of inappropriate antibiotic use.

Students in this study also reported the use of ivermectin, a drug that gained popularity due to speculative claims regarding its efficacy against COVID-19. Although its usage was not as high as that of antibiotics, its presence in the students’ self-medication habits illustrates the extent to which misinformation influenced pharmaceutical choices. Similarly, chloroquine, despite being minimally used, was still reported. The consumption of vitamins, ibuprofen, and other over-the-counter medications further exemplifies the diversity of drugs taken without professional guidance.

A key contribution of this study is the identification of the concurrent use of conventional medicines and herbal remedies for the prevention and treatment of COVID-19 among health students. While previous studies documented self-medication during the COVID-19 pandemic, most of them focused exclusively on conventional or herbal products separately. In the Mozambican context, where traditional medicine is widely practiced and culturally embedded, the dual use observed in this study may reflect a common practice. These findings underscore the need for educational reform and the integration of phytotherapy training into health curricula where it is currently absent, which could help students make informed, evidence-based decisions.

Despite providing relevant information on self-medication patterns among the study participants, this study presents some limitations. The use of a self-administered electronic questionnaire introduces potential response bias, as participants may have omitted or limited their answers, whether due to forgetfulness or social desirability. Additionally, the questionnaire was developed specifically for this study and was not formally pretested or piloted, which may have influenced participants’ interpretation of some questions. Furthermore, although a total population sampling strategy was adopted, the limited participation rate (16%) may have affected the representativeness of the findings. This response rate is comparable to similar online survey-based studies, particularly when no incentives are provided. While non-response bias cannot be ruled out, the study offers valuable preliminary evidence on self-medication practices among health students and highlights key areas for future investigation and intervention.

## Methods

### Study area and design

The study was conducted in Maputo, the capital and largest city of Mozambique, located on the western shore of Maputo Bay in the southernmost region of the country, near the borders with South Africa and Eswatini. Maputo is the main financial, corporate, and mercantile hub of Mozambique and constitutes both a municipality and a province.

A descriptive, cross-sectional study design was employed to obtain a snapshot of self-medication practices and herbal remedy use among health students in Maputo. This study is reported in accordance with the STROBE (Strengthening the Reporting of Observational Studies in Epidemiology) guidelines for cross-sectional studies^35^(Supplementary file 1).

### Study population

Undergraduate and graduate students enrolled in health-related programs at three major higher education institutions in Maputo: UEM, ISCISA, and ISCTEM, were recruited for this study. Inclusion criteria comprised students of all genders, aged 18 years or older, enrolled in programs leading to health professions defined by the World Health Organization^36^ and the International Labour Organization^37^. The exclusion criteria comprised students who had their enrollment canceled or suspended during the data collection period, those younger than 18 years of age, students enrolled in non-health programs, students who had already completed their studies or those who provided inconsistent information regarding their year of birth.

### Participant selection and data collection

Prior to data collection, the research team presented the study objectives to the administrative offices of the three selected institutions and obtained permission to conduct the research. Subsequently, the team conducted in-class visits to inform students enrolled in health-related programs about the upcoming survey. Following institutional approval, access was requested to the email and phone contact lists of students enrolled in health-related programs. Using a total population sampling strategy, all eligible students were invited to participate.

Data were collected through an online survey administered via Google Forms (http://forms.google.com/). The survey link, accompanied by an invitation to participate, was distributed through institutional contact lists, including emails and SMS from November 17 to 18, 2022. The survey remained open for responses for one month. Participants were informed about the study’s purpose, and those who agreed to participate provided informed consent before accessing the questionnaire (Supplementary file 2).

The electronic questionnaire was developed by the research team based on the study’s specific objectives and informed by prior experience in survey design and data collection in public health contexts. Designed to take approximately 10–15 min to complete, it included both closed and open-ended questions. The first section included demographic questions, followed by sections assessing participants’ perceptions and practices regarding the use of herbal remedies and conventional medicines, the types of conventional medicines and herbal remedies used and the sources of information.

The main outcome measures were: (i) the prevalence of self-medication for COVID-19 prevention or treatment among participants and (ii) the types of conventional medicines and herbal remedies used. In our study, self-medication was defined as the use of any herbal remedies or conventional medicines by participants for COVID-19. This included: (i) the use of herbal remedies for either prevention or treatment, and (ii) the use of conventional medicines specifically for prevention purposes. The logic of the survey was to capture use since the onset of the COVID-19 pandemic, as participants were asked whether they had ever used any of the reported remedies or medications for COVID-19.

### Data management and analysis

All responses were exported from Google forms into Microsoft Excel version 2019 and underwent a consistency check to identify inconsistencies and ineligible entries. Questionnaires with unclear or inconsistent birth year information were excluded according to predefined eligibility criteria. Partially completed questionnaires were retained when they contained sufficient information to determine self-medication status, whereas item-level omissions were kept as missing and are reported as such in the tables. Descriptive statistics were applied to calculate proportions and frequencies of plant species and medicines used. All analysis were performed using STATA version 14.2.

### Ethical considerations

This study was conducted in accordance with the Declaration of Helsinki. It followed the Checklist for Reporting Electronic Internet Search Results (CHERRIES) guidelines^38^(Supplementary file 3). Ethical approval was granted by the Eduardo Mondlane University and Maputo Central Hospital Joint Institutional Committee on Bioethics for Health (CIBS FM & HCM), with the protocol registered under number CIBS FM&HCM/011/2022. The study was authorized by each institution where data were collected. Participant confidentiality was ensured, and their information remained anonymous.

The study was introduced through a preliminary meeting between the researchers and administrative authorities of each of the three institutions (UEM, ISCTEM and ISCISA). The meeting aimed to inform and clarify all stakeholders regarding the objectives. An information note was distributed, and announcements were made in each class regarding the study. Participation was entirely voluntary, and no financial compensation, incentives, or other rewards were offered to participants for taking part in the study.

## Supplementary Information

Below is the link to the electronic supplementary material.

## Data Availability

The dataset generated during the current study is available in the Zenodo repository: https://doi.org/10.5281/zenodo.19951302.
