# Supplementary material for: Self-medication with herbal remedies and pharmaceuticals for COVID-19 among healthcare students in Maputo
Source: Sci Rep. 2026 Jun 16;16:27574. doi: 10.1038/s41598-026-53105-2 (PMC13538521; doi:10.1038/s41598-026-53105-2)
Supplement: Supplementary file 1 [file 41598_2026_53105_MOESM2_ESM.docx]

**Self-medication with herbal remedies and pharmaceuticals for COVID-19 among healthcare students in Maputo**

Esperança João Rafael^1^, Filomena Barbosa^1^, Saquina Rugunate^2^, Delfina Fernandes Hlashwayo^1*^

^1^ Department of Biological Sciences, Faculty of Sciences, Eduardo Mondlane University, Maputo, Mozambique

^2^ Instituto Superior de Ciências e Tecnologia de Moçambique

*Corresponding author: [delfina.hlashwayo@uem.ac.mz](mailto:delfina.hlashwayo@uem.ac.mz)

**Study Questionnaire**

*Note: Section 1 corresponds to the Participant Information Sheet and Informed Consent Form. Participants were required to read the information provided and indicate their informed consent before proceeding with the questionnaire.*

**SECTION 2: Socio-demographic data**

1. What health-related degree program are you enrolled in?

○ Medicine

○ Nursing

○ Dentistry

○ Pharmacy

○ Biology and health

○ Master’s in Public Health

○ Master’s in Biosciences

○ PhD in Biosciences and Public Health

○ Other _____________

2. Which institution do you attend?

○ UEM

○ ISCISA

○ ISCTEM

3. What year of study are you currently in?

○ 1^st^ year

○ 2^nd^ year

○ 3^rd^ year

○ 4^th^ year

○ 5^th^ year

○ 6^th^ year

○ Other ___________________

4. What is your date of birth?

Day, month, year ____, ____, _____

5. What is your gender?

○ Male

○ Female

6. What is your nationality?

○ Mozambican

○ Other ______________________

7. Which province are you originally from?

○ Niassa

○ Cabo Delgado

○ Nampula

○ Zambézia

○ Tete

○ Manica

○ Sofala

○ Gaza

○ Inhambane

○ Maputo province

○ Maputo (city)

○ Other _____________

8. Which municipal district do you currently reside in?

○ KaMpfumu Municipal District

○ Nhlamankulu Municipal District

○ KaMaxakeni Municipal District

○ KaMavota Municipal District

○ KaMubukwana Municipal District

○ KaTembe Municipal District

○ KaNyaka Municipal District

○ Other _____________

9. What is your marital status?

○ Single

○ Married

○ Divorced

○ Widowed

○ Other _____________

10. What is your religion?

○ Catholic

○ Protestant

○ Islamic

○ No religion

○ Other __________

11. Have you been diagnosed with a chronic disease (e.g., diabetes, cardiovascular disease, asthma, cancer, liver disease, kidney disease, etc.)?

○ Yes

○ No

○ I do not know

12. Since the beginning of the pandemic in 2020, have you experienced any COVID-19-related symptoms (fever, dry cough, difficulty breathing, tiredness, sore throat, etc.)?

○ Yes

○ No

13. Have you been vaccinated against COVID-19?

○ Yes, 1 dose

○ Yes, 2 doses

○ Yes, more than 2 doses

○ No

14. Have you been diagnosed with COVID-19 since the beginning of the pandemic?

○ Yes

○ No

*Note: If the response to Question 14 was “YES”, participants were directed to Section 3. If the response was “NO”, they were directed to Section 5.*

**SECTION 3: Use of pharmaceuticals for the treatment of COVID-19**

*In this section, we ask participants to answer questions about the use of pharmaceuticals to treat COVID-19.*

1. What was the clinical picture of the illness?

○ Asymptomatic

○ Mild (did not receive oxygen)

○ Moderate (did not receive oxygen, but had signs of non-severe pneumonia)

○ Severe (Required oxygen therapy or oxygen was indicated but unavailable)

○ Critical (Required invasive ventilation (or maximum available respiratory support))

2. Did you take any medication prescribed by a doctor to treat COVID-19?

○ No, but it was necessary

○ No, there was no need because I was asymptomatic

○ Yes

3. If you answered "Yes" to the previous question, which medication(s) did you take? (select all that apply)

□ Azithromycin

□ Amoxicillin

□ Ibuprofen

□ Paracetamol

□ Dexamethasone

□ Methylprednisolone

□ Chloroquine

□ Hydroxychloroquine

□ Ivermectin

□ Not applicable (I did not take any medication)

□ Other ___________________

**SECTION 4: Use of pharmaceuticals for COVID-19 prevention**

1. Have you taken any medication to prevent COVID-19?

○ Yes

○ No

2. If you answered "Yes" to the previous question, which medication(s) did you take? (select all that apply)

□ Azithromycin

□ Amoxicillin

□ Ibuprofen

□ Paracetamol

□ Dexamethasone

□ Methylprednisolone

□ Chloroquine

□ Hydroxychloroquine

□ Ivermectin

□ Not applicable (I did not take any medication)

□ Other ___________________

3. Have you ever used herbal remedies to prevent COVID-19?

*For the purposes of this study, ‘herbal remedies’ are defined as all plant-based remedies that are not based on isolated active compounds. For example: teas (e.g. lemon tea), eucalyptus leaf steam inhalation, traditional herbal remedies, and other plant-based remedies.*

○ Yes, for prevention and treatment

○ Yes, only for prevention

○ Yes, only for treatment

○ No

*Note: If the answer to Question 3 was “Yes, for prevention and treatment”, the form automatically proceeded to Section 6. If the answer was “Yes, only for prevention”, it automatically proceeded to Section 8. If the answer was “Yes, only for treatment”, it automatically proceeded to Section 7. If the answer was “No”, the survey ended.*

**SECTION 5: Use of pharmaceuticals for COVID-19 prevention**

*In this section, we ask participants to answer questions about the use of pharmaceuticals for COVID-19 prevention.*

1. Have you taken any medication to prevent COVID-19?

○ Yes

○ No

2. If you answered "Yes" to the previous question, which medication(s) did you take? (select all that apply)

□ Azithromycin

□ Amoxicillin

□ Ibuprofen

□ Paracetamol

□ Dexamethasone

□ Methylprednisolone

□ Chloroquine

□ Hydroxychloroquine

□ Ivermectin

□ Not applicable (I did not take any medication)

□ Other ___________________

3. Have you ever used herbal remedies to prevent COVID-19?

*For the purposes of this study, ‘herbal remedies’ are defined as all plant-based remedies that are not based on isolated active compounds. For example: teas (e.g. lemon tea), eucalyptus leaf steam inhalation, traditional herbal remedies, and other plant-based remedies.*

○ Yes

○ No

*Note: If the answer to Question 3 was “Yes”, the form automatically proceeded to Section 8. If the answer was “No”, the survey was terminated.*

**SECTION 6: Use of herbal remedies for COVID-19 treatment (participants who used herbal remedies for both prevention and treatment)**

1. What herbal remedies did you use to treat COVID-19? (select all that apply)

□ Eucalyptus leaf steam inhalation

□ Lemon leaf steam inhalation

□ Steam inhalation with eucalyptus and lemon leaves

□ Mango leaf steam inhalation

□ Onion syrup

□ Carrot syrup

□ Onions and carrot syrup mixed

□ Cactus syrup

□ Lemon tea

□ Ginger tea

□ Ginger and garlic tea

□ Raw garlic

□ Garlic tea

□ Guava leaves tea

□ Other______________________________________________

2. What were the sources of information? (select all that apply)

□ Doctor

□ TV

□ Newspaper

□ Social media (Facebook/Twitter/Instagram/WhatsApp)

□ Internet search (e.g., Google or other search engines)

□ Ministry of Health (MISAU) website

□ Books

□ Scientific articles

□ Family / friend / parent

□ Workshop / Seminars

□ Traditional healer

□ Other ________________

3. On a scale from 0 to 5, what was the effect of the herbal remedies on your recovery from COVID-19?

○ 0 (no effect) ○ 1	○ 2	○ 3	○ 4	○ 5 (great effect)

4. Which COVID-19 symptoms improved after using the herbal remedies?

________________________________

5. If you mentioned more than one remedy, which was the most effective?

____________________

6. Did you experience any side effects from the herbal remedies you used?

○ Yes

○ No

○ Other _________________

7. If you answered yes to the previous question, which remedy and what were the side effects?

_________________________________________________________

8. Have you used herbal remedies to treat any other illness in the last 12 months?

○ Yes

○ No

*Note: After Section 6, the form automatically proceeded to Section 8.*

**SECTION 7: Use of herbal remedies for COVID-19 treatment (participants who used herbal remedies for treatment only)**

1. What herbal remedies did you use to treat COVID-19? (select all that apply)

□ Eucalyptus leaf steam inhalation

□ Lemon leaf steam inhalation

□ Steam inhalation with eucalyptus and lemon leaves

□ Mango leaf steam inhalation

□ Onion syrup

□ Carrot syrup

□ Onions and carrot syrup mixed

□ Cactus syrup

□ Lemon tea

□ Ginger tea

□ Ginger and garlic tea

□ Raw garlic

□ Garlic tea

□ Guava leaves tea

□ Other______________________________________________

2. What were your sources of information? (select all that apply)

□ Doctor

□ TV

□ Newspaper

□ Social media (Facebook/Twitter/Instagram/WhatsApp)

□ Internet search (e.g., Google or other search engines)

□ Ministry of Health (MISAU) website

□ Books

□ Scientific articles

□ Family / friend / parent

□ Workshop / Seminars

□ Traditional healer

□ Other ________________

3. On a scale from 0 to 5, what was the effect of the herbal remedies on your recovery from COVID-19?

○ 0 (no effect) ○ 1	○ 2	○ 3	○ 4	○ 5 (great effect)

4. Which COVID-19 symptoms improved after using the herbal remedies?

________________________________

5. If you mentioned more than one remedy, which was the most effective?

____________________

6. Did you experience any side effects from the herbal remedies you used?

○ Yes

○ No

○ Other _________________

7. If you answered yes to the previous question, which remedy and what were the side effects?

_________________________________________________________

8. Have you used herbal remedies to treat any other illness in the last 12 months?

○ Yes

○ No

9. Do you have any additional information on the use of herbal remedies in the context of COVID-19 that you would like to share?

_________________________________________________________________________________________________

_________________________________________________________________________________________________

*Note: Upon completion of Section 7, the survey was automatically terminated.*

**SECTION 8: Use of herbal remedies for COVID-19 prevention**

1. What herbal remedies did you use to prevent COVID-19? (select all that apply)

□ Eucalyptus leaf steam inhalation

□ Lemon leaf steam inhalation

□ Steam inhalation with eucalyptus and lemon leaves

□ Mango leaf steam inhalation

□ Onion syrup

□ Carrot syrup

□ Onions and carrot syrup mixed

□ Cactus syrup

□ Lemon tea

□ Ginger tea

□ Ginger and garlic tea

□ Raw garlic

□ Garlic tea

□ Guava leaves tea

□ Other______________________________________________

2. What were your sources of information? (select all that apply)

□ Doctor

□ TV

□ Newspaper

□ Social media (Facebook/Twitter/Instagram/WhatsApp)

□ Internet search (e.g., Google or other search engines)

□ Ministry of Health (MISAU) website

□ Books

□ Scientific articles

□ Family / friend / parent

□ Workshop / Seminars

□ Traditional healer

□ Other ________________

3. For how long did you use herbal remedies?

○ Only once
○ For 1 month
○ For 3 months
○ For 6 months
○ For 1 year
○ Other _____________

4. How often did you use them?

○ Daily

○ Once a week

○ Once a month

○ Other ______________

5. Are you currently using them?

○ Yes

○ No

6. Have you used herbal remedies to prevent any other illness in the last 12 months?

○ Yes

○ No

7. Do you have any additional information on the use of herbal remedies in the context of COVID-19 that you would like to share?

_________________________________________________________________________________________________

_________________________________________________________________________________________________

_________________________________________________________________________________________________

***End of form***
