# Supplementary material for: Self-medication with herbal remedies and pharmaceuticals for COVID-19 among healthcare students in Maputo
Source: Sci Rep. 2026 Jun 16;16:27574. doi: 10.1038/s41598-026-53105-2 (PMC13538521; doi:10.1038/s41598-026-53105-2)
Supplement: Supplementary file 2 [file 41598_2026_53105_MOESM3_ESM.docx]

**Self-medication with herbal remedies and pharmaceuticals for COVID-19 among healthcare students in Maputo**

Esperança João Rafael^1^, Filomena Barbosa^1^, Saquina Rugunate^2^, Delfina Fernandes Hlashwayo^1*^

^1^ Department of Biological Sciences, Faculty of Sciences, Eduardo Mondlane University, Maputo, Mozambique

^2^ Instituto Superior de Ciências e Tecnologia de Moçambique

*Corresponding author: [delfina.hlashwayo@uem.ac.mz](mailto:delfina.hlashwayo@uem.ac.mz)

**Checklist for Reporting Results of Internet E-Surveys (CHERRIES)**

| ***Checklist Item*** | ***Explanation*** | ***Response*** | ***Location where item is reported*** |
| --- | --- | --- | --- |
| **Design** | | |  |
| Describe survey design | Describe target population, sample frame. Is the sample a convenience sample? (In “open” surveys this is most likely.) | Cross-sectional online survey targeting undergraduate and graduate students enrolled in health-related programs at three higher education institutions in Maputo (UEM, ISCISA, ISCTEM). A total population sampling approach was used, whereby all eligible students were invited to participate. | Methods – Study population & Participant selection and data collection |
| **IRB (Institutional Review Board) approval and informed consent process** | | | |
| IRB approval | Mention whether the study has been approved by an IRB. | Ethical approval was granted by the Eduardo Mondlane University and Maputo Central Hospital joint Institutional Committee on Bioethics for Health (CIBS FM & HCM), protocol number CIBS FM&HCM/011/2022. | Methods – Ethical considerations |
| Informed consent | Describe the informed consent process. Where were the participants told the length of time of the survey, which data were stored and where and for how long, who the investigator was, and the purpose of the study? | Participants were informed about the study objectives, data handling, investigator identity, and survey duration (10–15 minutes). Informed consent was obtained electronically before accessing the questionnaire. | Methods – Participant selection and data collection |
| Data protection | If any personal information was collected or stored, describe what mechanisms were used to protect unauthorized access. | Only non-identifiable data were collected; all responses were stored securely in Google Forms and exported to Microsoft Excel 2019 for analysis. Access was limited to the research team. | Methods – Data management and analysis |
| **Development and pre-testing** | | | |
| Development and testing | State how the survey was developed, including whether the usability and technical functionality of the electronic questionnaire had been tested before fielding the questionnaire. | Questionnaire was developed by the research team based on the study objectives and previous survey experience. No pretesting or pilot testing was conducted. | Methods – Participant selection and data collection |
| **Recruitment process and description of the sample having access to the questionnaire** | | | |
| Open survey versus closed survey | An “open survey” is a survey open for each visitor of a site, while a closed survey is only open to a sample which the investigator knows (password-protected survey). | The questionnaire was accessible via a survey link that was distributed only to students through institutional email and phone contact lists. While anyone with the link could technically access it, distribution was limited to eligible participants, minimizing unauthorized responses. | Methods – Participant selection and data collection |
| Contact mode | Indicate whether or not the initial contact with the potential participants was made on the Internet. (Investigators may also send out questionnaires by mail and allow for Web-based data entry.) | Initial contact with potential participants was made via in-class visits and online through email and SMS. Students were informed about the survey’s voluntary nature, purpose, and confidentiality, and invited to complete the online questionnaire via a link. | Methods – Participant selection and data collection |
| Advertising the survey | How/where was the survey announced or advertised? Some examples are offline media (newspapers), or online (mailing lists – If yes, which ones?) or banner ads (Where were these banner ads posted and what did they look like?). It is important to know the wording of the announcement as it will heavily influence who chooses to participate. Ideally the survey announcement should be published as an appendix. | The survey was announced during in-class visits, where the research team explained the study objectives, voluntary participation, and confidentiality of responses. Additionally, invitations with the survey link were sent via institutional email and SMS to all eligible students. | Methods – Participant selection and data collection |
| **Survey administration** | | | |
| Web/E-mail | State the type of e-survey (eg, one posted on a Web site, or one sent out through e-mail). If it is an e-mail survey, were the responses entered manually into a database, or was there an automatic method for capturing responses? | The survey was sent via institutional email and SMS with a Google Forms link. Responses were automatically captured in Google Forms and later exported to Microsoft Excel for consistency checks. | Methods – Participant selection and data collection; Data management and analysis |
| Context | Describe the Web site (for mailing list/newsgroup) in which the survey was posted. What is the Web site about, who is visiting it, what are visitors normally looking for? Discuss to what degree the content of the Web site could pre-select the sample or influence the results. For example, a survey about vaccination on a anti-immunization Web site will have different results from a Web survey conducted on a government Web site | The survey was sent directly to students enrolled in health-related programs at UEM, ISCISA, and ISCTEM. The findings are specific to this population and may not be generalizable to all Mozambican students. | Methods – Participant selection and data collection |
| Mandatory/voluntary | Was it a mandatory survey to be filled in by every visitor who wanted to enter the Web site, or was it a voluntary survey? | Participation was voluntary. Students were invited but could choose whether or not to complete the survey. | Methods – Participant selection and data collection |
| Incentives | Were any incentives offered (eg, monetary, prizes, or non-monetary incentives such as an offer to provide the survey results)? | No incentives were offered for participation. | Methods – Ethical considerations |
| Time/Date | In what timeframe were the data collected? | Invitations to participate in the survey were sent on November 17 and 18, 2022. The survey remained open for responses for one month. | Methods – Participant selection and data collection |
| Randomization of items or questionnaires | To prevent biases items can be randomized or alternated. | Items were presented in a fixed order; no randomization was applied. | Methods – Participant selection and data collection |
| Adaptive questioning | Use adaptive questioning (certain items, or only conditionally displayed based on responses to other items) to reduce number and complexity of the questions. | Adaptive questioning was implemented: after completing the sociodemographic section, participants were asked whether they had ever been diagnosed with COVID-19 since the start of the pandemic. Respondents who answered “yes” were directed to a section on the use of medicines for COVID-19 treatment, followed by sections on preventive medicine use and herbal remedies for treatment or prevention. Participants who answered “no” were directed to sections on the use of medicines for prevention and herbal remedies for prevention. | Methods – Participant selection and data collection; Supplementary material 2 |
| Number of Items | What was the number of questionnaire items per page? The number of items is an important factor for the completion rate. | The questionnaire included 14 sociodemographic items on the first section. In subsequent sections, depending on the participant’s responses regarding COVID-19 diagnosis, sections contained between 3 and 9 items. All items were displayed on a single Google Forms page per section. | Supplementary material 2 |
| Number of screens (pages) | Over how many pages was the questionnaire distributed? The number of items is an important factor for the completion rate. | The questionnaire was structured in 8 sections. All items within a section were displayed on a single Google Forms page. Depending on participants’ responses, some sections were conditionally shown, so not all participants viewed every section. | Supplementary material 2 |
| Completeness check | It is technically possible to do consistency or completeness checks before the questionnaire is submitted. Was this done, and if “yes”, how (usually JAVAScript)? An alternative is to check for completeness after the questionnaire has been submitted (and highlight mandatory items). If this has been done, it should be reported. All items should provide a non-response option such as “not applicable” or “rather not say”, and selection of one response option should be enforced. | Some items were mandatory in Google Forms, preventing submission if unanswered. Partially completed questionnaires were reviewed manually for eligibility, with missing responses reported as such. | Methods – Data management and analysis |
| Review step | State whether respondents were able to review and change their answers (eg, through a Back button or a Review step which displays a summary of the responses and asks the respondents if they are correct). | Participants were able to review and modify their responses before submission, as provided by the Google Forms interface. | Not reported in the manuscript (survey platform feature) |
| **Response rates** | | | |
| Unique site visitor | If you provide view rates or participation rates, you need to define how you determined a unique visitor. There are different techniques available, based on IP addresses or cookies or both. | Unique participants were identified using combinations of sociodemographic characteristics. Although the procedure was planned to detect potential duplicates, no duplicate entries were observed. | Not explicitly reported in the manuscript; provided here in accordance with CHERRIES reporting requirements. |
| View rate (Ratio of unique survey visitors/unique site visitors) | Requires counting unique visitors to the first page of the survey, divided by the number of unique site visitors (not page views!). It is not unusual to have view rates of less than 0.1 % if the survey is voluntary. | Not applicable; the survey was conducted via Google Forms, which does not provide information on unique site visitors. | Not explicitly reported in the manuscript; provided here in accordance with CHERRIES reporting requirements. |
| Participation rate (Ratio of unique visitors who agreed to participate/unique first survey page visitors) | Count the unique number of people who filled in the first survey page (or agreed to participate, for example by checking a checkbox), divided by visitors who visit the first page of the survey (or the informed consents page, if present). This can also be called “recruitment” rate. | 444 students agreed to participate after accessing the survey link, out of 2,788 invited, yielding a participation rate of approximately 16%. | Results – Sociodemographic characteristics of study participants |
| Completion rate (Ratio of users who finished the survey/users who agreed to participate) | The number of people submitting the last questionnaire page, divided by the number of people who agreed to participate (or submitted the first survey page). This is only relevant if there is a separate “informed consent” page or if the survey goes over several pages. This is a measure for attrition. Note that “completion” can involve leaving questionnaire items blank. This is not a measure for how completely questionnaires were filled in. (If you need a measure for this, use the word “completeness rate”.) | 100% of participants who provided informed consent completed the survey. | Results – Sociodemographic characteristics of study participants |
| **Preventing multiple entries from the same individual** | | | |
| Cookies used | Indicate whether cookies were used to assign a unique user identifier to each client computer. If so, mention the page on which the cookie was set and read, and how long the cookie was valid. Were duplicate entries avoided by preventing users access to the survey twice; or were duplicate database entries having the same user ID eliminated before analysis? In the latter case, which entries were kept for analysis (eg, the first entry or the most recent)? | No browser cookies were used to assign unique identifiers or prevent multiple entries. Potential duplicate entries were assessed post hoc using combinations of sociodemographic variables (course, institution, date of birth, sex, district/municipality, marital status, and religion), but no duplicates were identified. All submitted entries were retained for analysis. | Not explicitly reported in the manuscript; provided here in accordance with CHERRIES reporting requirements. |
| IP check | Indicate whether the IP address of the client computer was used to identify potential duplicate entries from the same user. If so, mention the period of time for which no two entries from the same IP address were allowed (eg, 24 hours). Were duplicate entries avoided by preventing users with the same IP address access to the survey twice; or were duplicate database entries having the same IP address within a given period of time eliminated before analysis? If the latter, which entries were kept for analysis (eg, the first entry or the most recent)? | The IP address of participants’ devices was not used to identify or prevent potential duplicate entries. | Not explicitly reported in the manuscript; provided here in accordance with CHERRIES reporting requirements. |
| Log file analysis | Indicate whether other techniques to analyze the log file for identification of multiple entries were used. If so, please describe. | No additional log file analysis techniques were used to identify multiple entries. | Not explicitly reported in the manuscript; provided here in accordance with CHERRIES reporting requirements. |
| Registration | In “closed” (non-open) surveys, users need to login first and it is easier to prevent duplicate entries from the same user. Describe how this was done. For example, was the survey never displayed a second time once the user had filled it in, or was the username stored together with the survey results and later eliminated? If the latter, which entries were kept for analysis (eg, the first entry or the most recent)? | Not applicable, as the survey did not require user login. | Not explicitly reported in the manuscript; provided here in accordance with CHERRIES reporting requirements. |
| **Analysis** | | | |
| Handling of incomplete questionnaires | Were only completed questionnaires analyzed? Were questionnaires which terminated early (where, for example, users did not go through all questionnaire pages) also analyzed? | Partially completed questionnaires were retained if they contained sufficient information to determine self-medication status. Item-level omissions were treated as missing and reported as such in the tables. Only questionnaires that were completely invalid (e.g., inconsistent birth year or failing eligibility criteria) were excluded. | Methods – Data management and analysis |
| Questionnaires submitted with an atypical timestamp | Some investigators may measure the time people needed to fill in a questionnaire and exclude questionnaires that were submitted too soon. Specify the timeframe that was used as a cut-off point, and describe how this point was determined. | No exclusion was based on completion time. All responses were considered valid if eligibility criteria were met, regardless of the time taken to complete the questionnaire. | Not explicitly reported in the manuscript; provided here in accordance with CHERRIES reporting requirements. |
| Statistical correction | Indicate whether any methods such as weighting of items or propensity scores have been used to adjust for the non-representative sample; if so, please describe the methods. | No statistical correction (e.g., weighting or propensity scores) was applied. Descriptive statistics were used to summarize participant characteristics and self-medication practices. | Not explicitly reported in the manuscript; provided here in accordance with CHERRIES reporting requirements. |
